# Correlation of Thyroid Hormone Levels and Sensitivity With Metabolic Syndrome in Older Adults

**DOI:** 10.1111/1753-0407.70208

**Published:** 2026-03-20

**Authors:** Fengjuan Hu, Hu Liu, Linlin Gu, Shuli Jia, Lixing Zhou, Wanyu Zhao, Xiaolei Liu, Birong Dong

**Affiliations:** ^1^ Center of Gerontology and Geriatrics, National Clinical Research Center for Geriatrics West China Hospital, Sichuan University Chengdu Sichuan China; ^2^ Department of Pathology West China Hospital, Sichuan University Chengdu Sichuan China; ^3^ Department of General Surgery West China Hospital, Sichuan University Chengdu China; ^4^ Liver Transplant Center, Transplant Center West China Hospital, Sichuan University Chengdu China; ^5^ Endocrine Metabolism Department and Geriatric Department Seventh People's Hospital of Chengdu Chengdu China

**Keywords:** hormone sensitivity, metabolic syndrome, older adults, thyroid hormone

## Abstract

**Objective:**

Previous studies have shown that thyroid function is associated with metabolic syndrome (MetS), but this association remains poorly understood in older adults. This study aimed to explore the relationships between thyroid hormone levels and sensitivity with MetS and its components in older adults.

**Methods:**

The 2018 baseline data of the West China Health and Aging Trends study were used. MetS was diagnosed according to the International Diabetes Federation definition. Central thyroid hormone sensitivity was assessed by the TSH Index (TSHI), Thyrotrophic T4 Resistance Index (TT4RI), Thyroid Feedback Quantile‐based Index (TFQI), and Parametric TFQI (PTFQI). Peripheral thyroid hormone sensitivity was assessed by the FT3 to FT4 ratio (FT3/FT4 ratio).

**Results:**

A total of 3796 participants were included. The prevalence of MetS was 40.31%. High FT3 levels and FT3/FT4 ratios were associated with abdominal obesity and hypertension; decreased FT3 levels were associated with hyperglycemia, hypertriglyceridemia and low HDL‐C; low FT4 levels were associated with abdominal obesity and hypertriglyceridemia; increased TSH, TSHI and TT4RI levels were associated with hypertriglyceridemia and hypertension; and increased TFQI and PTFQI were associated with hypertension (*p* < 0.05, for all). Increased FT3 (OR = 1.13, 95% CI: 1.05–1.22), TSH (OR = 1.12, 95% CI: 1.04–1.20), TSHI (OR = 1.09, 95% CI: 1.01–1.17), TT4RI (OR = 1.09, 95% CI: 1.01–1.16), FT3/FT4 (OR = 1.25, 95% CI: 1.16–1.35) and low FT4 (OR = 0.87, 95% CI: 0.81–0.94) were associated with MetS.

**Conclusion:**

Thyroid hormone levels are correlated with MetS and its components. Reduced central thyroid hormone sensitivity and increased peripheral thyroid hormone sensitivity are risk factors for MetS in older adults.

## Highlights

- Thyroid hormone levels are associated with MetS and its components.
- Impaired central thyroid hormone sensitivity is a risk factor for MetS and its components.
- An increased FT3/FT4 ratio is related to MetS and its components.

## Graphical abstract

In this cross‐sectional analysis of 3796 older adults (≥ 60 years) from the WCHAT study, reduced central (TSHI↑, TT4RI↑) and increased peripheral (FT3/FT4↑) thyroid hormone sensitivity were significantly associated with a higher risk of Metabolic Syndrome (MetS).

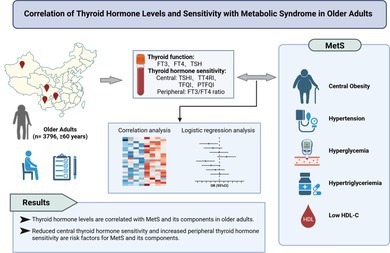

**Abbreviations**
ADL: activities of daily living
BMI: body mass index
BP: blood pressure
CHARLS: China Health and Retirement Longitudinal Study
CI: confidence interval
DBP: diastolic blood pressure
FPG: fasting plasma glucose
FT3: free triiodothyronine
FT3/FT4: FT3 to FT4 ratio
FT4: free tetraiodothyronine
HDL‐C: high‐density lipoprotein cholesterol
IR: insulin resistance
MetS: metabolic syndrome
OR: odds ratio
PTFQI: parametric thyroid feedback quantile‐based index
Q: quantile
SBP: systolic blood pressure
TFQI: thyroid feedback quantile‐based index
TG: triglycerides
TSH: thyroid‐stimulating hormone
TSHI: TSH index
TT4RI: thyrotrophic T4 resistance index
WC: waist circumference
WCHAT: West China Health and Aging Trend

## 1 Introduction

With the global aging population and changes in lifestyle, noncommunicable metabolic‐related diseases have become a major global public health challenge [1]. China, with the largest and fastest‐aging population, faces a particularly pressing burden. It is estimated that by 2050, people aged 65 and above will account for a quarter of the country's total population [2]. Rapid aging places a considerable burden on individuals with metabolic diseases, and there is an urgent need to pay attention to metabolic‐related diseases and their related factors in older adults.

Metabolic syndrome (MetS), as defined by the International Diabetes Federation (IDF), comprises a cluster of metabolic abnormalities, including central obesity, hypertension, hyperglycemia, and dyslipidemia. Globally, approximately one quarter of the population suffers from MetS [3]. Data from the China Health and Retirement Longitudinal Study (CHARLS) revealed that the prevalence of MetS among adults aged 60 years and above in China exceeded 30% by 2020 [4]. Given its strong association with cardiogenic and all‐cause mortality, understanding MetS risk factors is critical for improving prevention, screening, and systematic personalized intervention strategies [5].

The pathophysiology of MetS is not fully understood, although several mechanisms have been proposed, including insulin resistance (IR), chronic low‐grade inflammation, lipid toxicity, oxidative stress, genetic and epigenetic modifications, circadian rhythm disruption, and gut microbiota dysbiosis [6]. Furthermore, increasing evidence suggests a link between thyroid hormone levels/sensitivity and MetS. Both hyperthyroidism and overt/subclinical hypothyroidism can significantly affect metabolic regulation [7]. However, hyperthyroidism also leads to weight loss and improved lipid profiles, which may offset some aspects of MetS, making the relationship less clear. While several studies have identified a positive association between elevated thyroid‐stimulating hormone (TSH) levels and MetS risk [8, 9, 10], other studies from Taiwan and Tehran have failed to replicate this association [11, 12]. Similarly, although a high free triiodothyronine to free tetraiodothyronine (FT3/FT4) ratio has been proposed as a predictor of MetS [13, 14], the results from two cross‐sectional studies based on epidemiological and genetic analyses in the Chinese euthyroid population suggested an inverse relationship [7, 15]. Conflicting conclusions also exist regarding free tetraiodothyronine (FT4) [16, 17]. This makes it difficult to draw uniform conclusions from currently available studies. Additionally, studies conducted on older adults are rare. These discrepancies highlight the need for further investigation, particularly in older adults.

Therefore, this study aimed to elucidate the associations between thyroid hormone levels, thyroid hormone sensitivity, and MetS or its components in community‐dwelling older adults.

## 2 Materials and Methods

### 2.1 Study Design and Participants

The participants in this study were drawn from the baseline survey of the Western China Trends in Health and Aging (WCHAT) study. The detailed study design has been described elsewhere [18]. Briefly, the WCHAT study is a multicenter prospective cohort study conducted in Sichuan, Yunnan, Guizhou, and Xinjiang in western China. Well‐trained volunteers conducted face‐to‐face interviews and anthropometric measurements with participants at local hospitals or their community centers. Biological samples included fasting venous blood samples, saliva, urine, and feces.

**FIGURE 1:**
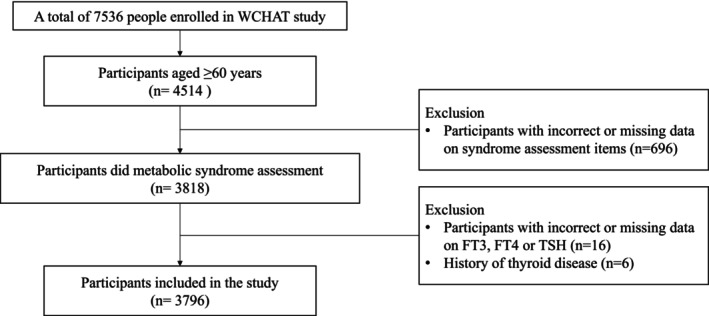
Study flow.

In this study, 4514 participants aged 60 years and older in the 2018 baseline were included. We subsequently excluded individuals for whom the MetS assessment was unavailable (*n* = 688) and those with incorrect data (*n* = 8). Sixteen participants with incorrect or missing data on FT3, FT4, or TSH were also excluded. Patients with a history of thyroid disease, including thyroid nodules, hyperthyroidism, hypothyroidism, goiter, and thyroid tumors, were further excluded (*n* = 6). Finally, 3796 participants aged 60 years and older were included in the final analysis. The detailed selection of the study population is shown in Figure 1.

### 2.2 Data Collection and Laboratory Examinations

The sociodemographic characteristics of all the study participants, including age, gender, ethnicity, education (illiterate/elementary school/middle school/high school and above), smoking history, alcohol consumption, physical activity, activities of daily living, and self‐reported chronic disease diagnoses, were assessed via a questionnaire. Anthropometric data, including height, weight, waist circumference (WC), and blood pressure (BP), were measured by trained technicians. The WC was measured midway between the lower border of the rib margin and the iliac crest with a flexible inelastic tape measure in the standing position. BP was measured twice consecutively while the participants were in a seated position with at least 5 min of rest between measurements, and the higher value was used in our analysis.

Fasting blood samples from the antecubital vein were obtained after an overnight fast. The samples were stored as whole blood and centrifuged at 3000 rpm for 10 min to obtain the serum. Biomarkers, including free triiodothyronine (FT3), FT4, TSH, fasting plasma glucose (FPG), triglyceride (TG), and high‐density lipoprotein cholesterol (HDL‐C), were detected.

### 2.3 Metabolic Syndrome Assessment

MetS was defined via the IDF definition [19]. An individual was categorized as having MetS if he/she had central obesity (WC ≥ 80 cm in women, ≥ 90 cm in men) plus any 2 of the following metabolic disorder components: (1) hypertension: systolic blood pressure (SBP) ≥ 130 mmHg and/or diastolic blood pressure (DBP) ≥ 85 mmHg, or previously diagnosed hypertension; (2) hyperglycemia: FPG ≥ 100.0 mg/dL (5.6 mmol/L), or previously diagnosed diabetes; (3) low HDL‐C: HDL‐C < 50 mg/dL (1.29 mmol/L) in women, < 40 mg/dL (1.03 mmol/L) in men; (4) hypertriglyceridemia: TG ≥ 150 mg/dL (1.7 mmol/L).

### 2.4 Assessment of Thyroid Function and Indices of Thyroid Hormone Sensitivity

Thyroid function was assessed by the levels of thyroid hormones, including TSH, FT4, and FT3. The indices of thyroid hormone sensitivity, including the TSH Index (TSHI), Thyrotrophic T4 Resistance Index (TT4RI), Thyroid Feedback Quantile‐based Index (TFQI), Parametric TFQI (PTFQI), and FT3/FT4 ratio, were calculated according to the following formulas [7, 20, 21, 22].

- TSHI = ln TSH (mIU/L) + 0.1345 × FT4 (pmol/L).
- TT4RI = FT4 (pmol/L) × TSH (mIU/L).
- TFQI = cdfFT4 − (1 − cdfTSH).
- PTFQI = NORM. DIST (FT_4_‐cell, μ FT4, σ FT4, TRUE) + NORM. DIST [ln (TSH‐cell), μ ln TSH, σ ln TSH, TRUE] – 1.
- FT3/FT4 ratio = FT3 (pmol/L)/FT4 (pmol/L).

In this study, μ FT4, σ FT4, μ Ln TSH, and σ ln TSH were 17.4273, 3.03355, 0.926183, and 0.795896, respectively. For TSHI and TT4RI, the higher the values are, the lower the central sensitivity to thyroid hormones is [7]. For the TFQI and PTFQI, negative values indicate higher central sensitivity to thyroid hormones, whereas positive values indicate lower sensitivity to thyroid hormones; a value of 0 indicates normal sensitivity [20, 21]. A higher FT3/FT4 ratio indicates higher peripheral thyroid hormone activity.

### 2.5 Statistical Analysis

Continuous variables are expressed as the mean ± standard deviation (SD). Categorical variables are shown as numbers (percentages). The independent sample *t*‐test was used for comparisons between groups for normally distributed continuous variables, and the nonparametric Mann–Whitney U test was used for non‐normally distributed variables. For categorical indicators, chi‐square tests were used to assess differences between groups.

The values of TSH and TT4RI were normalized for skewed distributions via logarithmic (ln) transformation. Multiple linear regression analysis was used to evaluate the relationships between thyroid function parameters and MetS components. Additionally, multivariate logistic regression models were constructed to evaluate the associations between thyroid hormone indices and MetS and its components. To explore these associations further, thyroid hormone indices were divided into quartiles or quintiles, and their associations with MetS were examined via logistic regression analysis. Potential confounders affecting MetS and its components were adjusted for in two sequential models: Model 1 accounted for age and gender, and Model 2 was additionally adjusted for body mass index (BMI), race, smoking status, alcohol consumption and comorbidity burden. All the statistical analyses were conducted via Stata version 16.0 (Stata Corp, College Station, TX, USA). A two‐sided *p* value < 0.05 was considered statistically significant.

## 3 Results

### 3.1 Baseline Characteristics

This study included 1574 (41.46%) men and 2222 (58.54%) women aged ≥ 60 years. Table 1 shows the baseline characteristics of the participants. Compared with the non‐Mets group, the Mets group was significantly younger and had a higher BMI (all *p* < 0.001). There were also significant differences among the groups in terms of gender, ethnicity, smoking status, alcohol consumption, and comorbidity burden (all *p* < 0.001). In addition, the prevalence of hypertension (*p* < 0.001), diabetes (*p* < 0.001), coronary heart disease (*p* < 0.001), and cerebrovascular disease (*p* = 0.006) significantly increased in the MetS group. There were no differences in education or physical activity levels.

**TABLE 1:** Baseline characteristics of study participants (*N* = 3796).

Characteristics	Overall	MetS (*n* = 1530)	Non‐MetS (*n* = 2266)	*p* value
Age, mean (SD)	67.78 (5.96)	67.26 (5.61)	68.13 (6.16)	< 0.001
Gender, *N* (%)				< 0.001
Male	1574 (41.46)	409 (26.73)	1165 (51.41)	
Female	2222 (58.54)	1121 (73.27)	1101 (48.59)	
BMI, mean (SD)	25.10 (4.11)	27.18 (3.55)	23.69 (3.86)	< 0.001
Ethnicity, *N* (%)				< 0.001
Han	1486 (39.15)	618 (40.39)	868 (38.31)	
Tibetan	661 (17.41)	246 (16.08)	415 (18.31)	
Qiang	845 (22.26)	375 (24.51)	470 (20.74)	
Yi	336 (8.85)	92 (6.01)	244 (10.77)	
Others	468 (12.33)	199 (13.01)	269 (11.87)	
Education, *N* (%)				0.080
Illiterate	1297 (34.18)	538 (35.16)	759 (33.51)	
Elementary	1439 (37.92)	560 (36.60)	879 (38.81)	
Middle	589 (15.52)	223 (14.58)	366 (16.16)	
High and above	470 (12.38)	209 (13.66)	261 (11.52)	
Smoking history, *N* (%)				< 0.001
Yes	783 (20.76)	194 (12.74)	589 (26.20)	
No	2988 (79.24)	1329 (87.26)	1659 (73.80)	
Drinking alcohol, *N* (%)				< 0.001
Yes	1041 (27.48)	342 (22.37)	699 (30.94)	
No	2747 (72.52)	1187 (77.63)	1560 (69.06)	
Physical activity, *N* (%)				0.051
Low	818 (21.60)	306 (20.01)	512 (22.67)	
Moderate to high	2969 (78.40)	1223 (79.99)	1746 (77.33)	
Hypertension				< 0.001
Yes	1145 (30.17)	630 (41.18)	515 (22.74)	
No	2650 (69.83)	900 (58.82)	1750 (77.26)	
Diabetes				< 0.001
Yes	325 (8.56)	231 (15.10)	94 (4.15)	
No	3470 (91.44)	1299 (84.90)	2171 (95.85)	
Coronary heart disease				< 0.001
Yes	201 (5.30)	106 (6.93)	95 (4.19)	
No	3594 (94.70)	1424 (93.07)	2170 (95.81)	
Cerebrovascular diseases				0.006
Yes	101 (2.66)	54 (3.53)	47 (2.08)	
No	3694 (97.34)	1476 (96.47)	2218 (97.92)	
Comorbidity burden				< 0.001
0	2014 (53.07)	671 (43.86)	1343 (59.29)	
1–2	1453 (38.29)	666 (43.53)	787 (34.75)	
≥ 3	328 (8.64)	193 (12.61)	135 (5.96)	
ADL score				0.614
100	3306 (87.28)	1326 (86.78)	1980 (87.61)	
65–95	475 (12.54)	199 (13.02)	276 (12.21)	
45–60	4 (0.11)	1 (0.07)	3 (0.13)	
≤ 40	3 (0.08)	2 (0.13)	1 (0.04)	
WC (cm)	87.28 (11.24)	94.22 (8.46)	82.59 (10.42)	< 0.001
SBP (mmHg)	137.19 (21.44)	143.90 (20.27)	132.65 (21.02)	< 0.001
DBP (mmHg)	82.78 (12.54)	85.44 (12.03)	80.99 (12.56)	< 0.001
FPG (mmol/L)	5.64 (1.78)	6.18 (2.11)	5.27 (1.40)	< 0.001
HDL‐C (mmol/L)	1.35 (0.38)	1.20 (0.29)	1.45 (0.40)	< 0.001
TG (mmol/L)	1.79 (1.51)	2.46 (1.93)	1.34 (0.90)	< 0.001
FT3 (pmol/L)	4.53 (0.77)	4.55 (0.78)	4.52 (0.76)	0.281
FT4 (pmol/L)	17.51 (2.82)	17.30 (2.78)	17.65 (2.84)	< 0.001
TSH (mIU/L)	3.46 (3.36)	3.75 (3.72)	3.27 (3.08)	< 0.001
TFQI	−0.02 (0.30)	−0.01 (0.28)	−0.02 (0.31)	0.902
PTFQI	0.03 (0.34)	0.04 (0.32)	0.02 (0.35)	0.220
TSHI	3.31 (0.78)	3.37 (0.73)	3.28 (0.81)	< 0.001
TT4RI	57.93 (45.10)	61.12 (46.48)	55.78 (44.03)	< 0.001
FT3/FT4	0.26 (0.06)	0.27 (0.06)	0.26 (0.05)	< 0.001

*Note:* Data are presented as mean (SD) or numbers (percentages).

Abbreviations: ADL, activities of daily living; BMI, body mass index; DBP, diastolic blood pressure; FPG, fasting plasma glucose; FT3/FT4, FT3 to FT4 ratio; FT3, free triiodothyronine; FT4, free tetraiodothyronine; HDL‐C, high‐density lipoprotein cholesterol; MetS, metabolic syndrome; PTFQI, parametric thyroid feedback quantile‐based index; SBP, systolic blood pressure; TFQI, thyroid feedback quantile‐based index; TG, triglycerides; TSH, thyroid‐stimulating hormone; TSHI, TSH index; TT4RI, thyrotrophic T4 resistance index; WC, waist circumference.

Indicators related to metabolism, including WC, SBP, DBP, FPG, and TG, were all significantly higher in MetS participants, whereas HDL‐C was significantly lower in MetS participants (all *p* < 0.001). In addition, significantly lower FT4 levels and higher TSH, TSHI, and TT4RI levels and FT3/FT4 ratios were detected in the MetS group than in the non‐MetS group (all *p* < 0.001). No differences were observed with respect to the FT3 levels or the TFQI and PTFQI values between the groups.

### 3.2 Associations of Thyroid Hormone Sensitivity Indices With MetS Parameters

Linear regression analysis revealed that after adjustment for age, gender, BMI (except high WC), race, smoking status, alcohol consumption, and comorbidity burden, FT3 was significantly associated with increased WC, high SBP, high DBP, decreased FPG, high HDL‐C, and low TG (Table 2). FT4 was significantly associated with decreased WC and low TG. Ln TSH was significantly associated with increased WC, high SBP, high DBP, low HDL‐C, and high TG. The TFQI score was significantly associated with high SBP and high DBP. The PTFQI was significantly associated with high SBP, high DBP, and low HDL‐C. TSHI was significantly associated with high SBP, high DBP, low HDL‐C, and high TG. Ln TT4RI was significantly associated with high SBP, high DBP, low HDL‐C, and high TG. FT3/FT4 was significantly associated with increased WC, high SBP, high DBP, and decreased FPG.

**TABLE 2:** Associations of thyroid hormones sensitivity indices with MetS parameters by linear regression.

MetS parameters	WC	SBP	DBP	FPG	HDL‐C	TG
Model 1						
FT3	1.53 (1.05 to 2.00)***	2.82 (1.91 to 3.72)***	1.18 (0.66 to 1.71)***	−0.15 (−0.23 to −0.08)***	0.01 (−0.00 to 0.03)	−0.10 (−0.16 to −0.03)**
FT4	−0.19 (−0.31 to 0.06)**	0.19 (−0.06 to 0.43)	0.11 (−0.03 to 0.25)	0.03 (0.01 to 0.05)*	−0.00 (−0.01 to 0.00)	−0.06 (−0.08 to −0.04)***
Ln TSH	0.63 (0.17 to 1.09)**	1.88 (1.01 to 2.75)***	1.24 (0.74 to 1.75)***	0.07 (−0.00 to 0.15)	−0.03 (−0.05 to −0.02)***	0.16 (0.10 to 0.22)***
TFQI	−0.40 (−1.59 to 0.80)	5.49 (3.22 to 7.76)***	3.45 (2.13 to 4.77)***	0.37 (0.18 to 0.56)***	−0.09 (−0.13 to −0.05)***	−0.20 (−0.36 to −0.04)*
PTFQI	−0.01 (−1.06 to 1.03)	5.24 (3.25 to 7.23)***	3.36 (2.20 to 4.51)***	0.34 (0.17 to 0.50)***	−0.10 (−0.13 to −0.06)***	−0.09 (−0.23 to 0.05)
TSHI	0.31 (−0.15 to 0.77)	2.20 (1.33 to 3.07)***	1.43 (0.93 to 1.94)***	0.12 (0.05 to 0.19)**	−0.04 (−0.06 to −0.03)***	0.06 (−0.01 to 0.12)
Ln TT4RI	0.51 (0.04 to 0.98)*	2.14 (1.25 to 3.04)***	1.40 (0.88 to 1.92)***	0.10 (0.02 to 0.17)*	−0.04 (−0.05 to −0.02)***	0.12 (0.05 to 0.18)***
FT3/FT4	24.04 (17.60 to 30.47)***	22.62 (10.27 to 34.96)***	9.01 (1.85 to 16.17)*	−2.65 (−3.68 to −1.62)***	0.30 (0.09 to 0.52)**	1.47 (0.60 to 2.33)**
Model 2						
FT3	1.52 (1.06 to 1.99)***	2.09 (1.20 to 2.98)***	0.96 (0.44 to 1.48)***	−0.16 (−0.23 to −0.08)***	0.02 (0.00 to 0.03)*	−0.15 (−0.21 to −0.09)***
FT4	−0.19 (−0.32‐ −0.06)**	0.15 (−0.09 to 0.39)	0.08 (−0.06 to 0.22)	0.01 (−0.01 to 0.03)	0.00 (−0.00 to 0.01)	−0.06 (−0.08 to −0.04)***
Ln TSH	0.56 (0.11 to 1.01)*	1.24 (0.38 to 2.09)**	0.89 (0.39 to 1.39)**	0.02 (−0.05 to 0.10)	−0.02 (−0.03 to −0.00)*	0.18 (0.12 to 0.24)***
TFQI	−0.83 (−2.04 to 0.39)	4.54 (2.23 to 6.85)***	2.86 (1.50 to 4.21)***	0.17 (−0.03 to 0.36)	−0.02 (−0.07 to 0.02)	−0.15 (−0.31 to 0.02)
PTFQI	−0.27 (−1.35 to 0.81)	4.21 (2.18 to 6.25)***	2.77 (1.57 to 3.97)***	0.15 (−0.02 to 0.32)	−0.04 (−0.07 to −0.00)*	−0.03 (−0.18 to 0.11)
TSHI	0.25 (−0.21 to 0.71)	1.57 (0.70 to 2.45)***	1.08 (0.56 to 1.60)***	0.05 (−0.03 to 0.12)	−0.02 (−0.03 to −0.00)*	0.08 (0.02 to 0.14)*
Ln TT4RI	0.45 (−0.02 to 0.92)	1.47 (0.58 to 2.36)**	1.02 (0.50 to 1.55)***	0.03 (−0.04 to 0.11)	−0.02 (−0.03 to −0.00)*	0.14 (0.08 to 0.21)***
FT3/FT4	26.18 (19.62 to 32.74)***	18.19 (5.51 to 30.88)**	9.16 (1.70 to 16.63)*	−2.21 (−3.28 to −1.13)***	0.12 (−0.10 to 0.34)	0.83 (−0.07 to 1.74)

*Note:* data are presented as *β* (95% CI). TSH and TT4RI were ln‐transformed for normal distribution before linear regression analysis. Model 1: adjusted for age, gender. Model 2: adjusted for age, gender, BMI (except in WC), race, smoking status, alcohol consumption and comorbidity burden. *p < 0.05; **p < 0.01; ***p < 0.001.

Abbreviations: DBP, diastolic blood pressure; FT3/FT4, FT3 to FT4 ratio; FT3, free triiodothyronine; FT4, free tetraiodothyronine; FPG, fasting plasma glucose; HDL‐C, high‐density lipoprotein cholesterol; MetS, metabolic syndrome; PTFQI, parametric thyroid feedback quantile‐based index; SBP, systolic blood pressure; TFQI, thyroid feedback quantile‐based index; TG, triglycerides; TSH, thyroid‐stimulating hormone; TT4RI: thyrotrophic T4 resistance index; TSHI, TSH index; WC, waist circumference.

**FIGURE 2:**
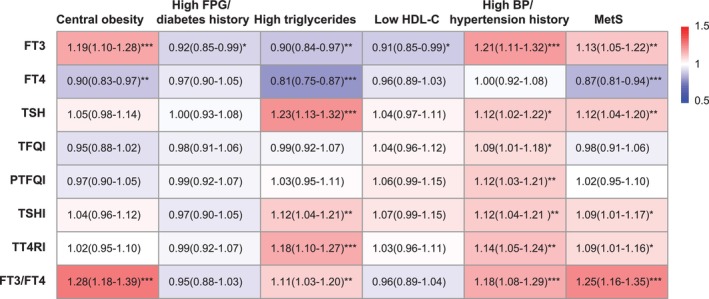
Association of thyroid hormone levels and sensitivity with MetS parameters by logistic regression. Data are presented as OR (95% CI). Age, gender, BMI (except in central obesity and MetS), race, smoking status, alcohol consumption and comorbidity burden were adjusted. *p < 0.05; **p < 0.01; ***p < 0.001. BMI, body mass index; BP, blood pressure; CI, confidence interval; FPG, fasting plasma glucose; FT3/FT4, FT3 to FT4 ratio; FT3, free triiodothyronine; FT4, free tetraiodothyronine; HDL‐C, high‐density lipoprotein cholesterol; MetS, metabolic syndrome; OR, odds ratio; PTFQI, parametric thyroid feedback quantile‐based index; TFQI, thyroid feedback quantile‐based index; TSH, thyroid‐stimulating hormone; TSHI, TSH index; TT4RI, thyrotrophic T4 resistance index.

Multiple logistic regression analyses between the thyroid hormone sensitivity indices and MetS parameters are shown in Figure 2. With every 1‐SD increase in FT3 levels, the odds ratios (ORs) for central obesity, hyperglycemia or diabetes, hypertriglyceridemia, low HDL‐C, hypertension, and MetS were 1.19 (95% CI: 1.10–1.28), 0.92 (95% CI: 0.85–0.99), 0.90 (95% CI: 0.84–0.97), 0.91 (95% CI: 0.85–0.99), 1.21 (95% CI: 1.11–1.32), and 1.13 (95% CI: 1.05–1.22), respectively. With every 1 SD increase in FT4 levels, the ORs for central obesity, hypertriglyceridemia, and MetS were 0.90 (95% CI: 0.83–0.97), 0.81 (95% CI: 0.75–0.87), and 0.87 (95% CI: 0.81–0.94), respectively. With every 1 SD increase in TSH levels, the ORs for hypertriglyceridemia, hypertension, and MetS were 1.23 (95% CI: 1.13–1.32), 1.12 (95% CI: 1.02–1.22), and 1.12 (95% CI: 1.04–1.20), respectively. With every 1‐SD increase in the TFQI and PTFQI, the ORs for hypertension were 1.09 (95% CI: 1.01–1.18) and 1.12 (95% CI: 1.03–1.21), respectively. With every 1 SD increase in TSHI, the ORs for hypertriglyceridemia, hypertension, and MetS were 1.12 (95% CI: 1.04–1.21), 1.12 (95% CI: 1.04–1.21), and 1.09 (95% CI: 1.01–1.17), respectively. With every 1 SD increase in TT4RI, the ORs for hypertriglyceridemia, hypertension, and MetS were 1.18 (95% CI: 1.10–1.27), 1.14 (95% CI: 1.05–1.24), and 1.09 (95% CI: 1.01–1.16), respectively. With every 1 SD increase in FT3/FT4 levels, the ORs for central obesity, hypertriglyceridemia, hypertension, and MetS were 1.28 (95% CI: 1.18–1.39), 1.11 (95% CI: 1.03–1.20), 1.18 (95% CI: 1.08–1.29), and 1.25 (95% CI: 1.16–1.35), respectively.

### 3.3 Associations of Thyroid Hormone Sensitivity Index Quartiles/Quintiles With the Risk of MetS

**FIGURE 3:**
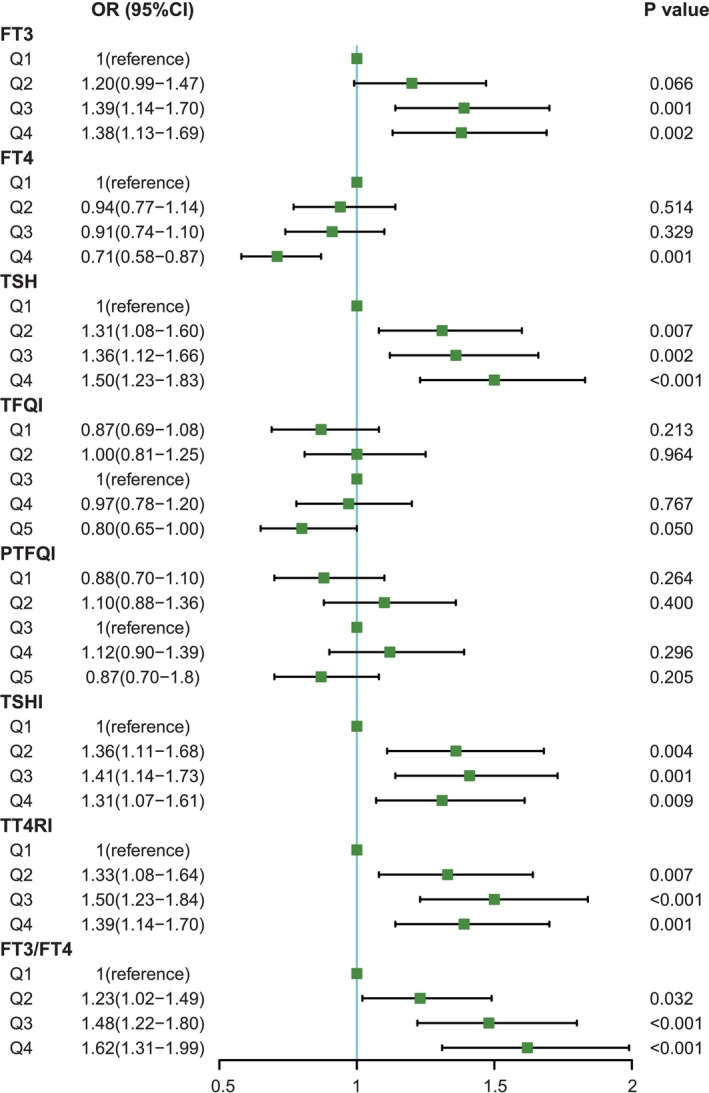
Association of thyroid hormone levels and sensitivity indices quartiles/quintiles with the risk of MetS. Data are presented as OR (95% CI). Age, gender, race, smoking status, alcohol consumption, and comorbidity burden were adjusted. CI, confidence interval; FT3/FT4, FT3 to FT4 ratio; FT3, free triiodothyronine; FT4, free tetraiodothyronine; MetS, metabolic syndrome; OR, odds ratio; PTFQI, Parametric Thyroid Feedback Quantile‐based Index; Q, quantile; TFQI, thyroid feedback quantile‐based index; TSH, thyroid‐stimulating hormone; TSHI, TSH index; TT4RI, thyrotrophic T4 resistance index.

When the thyroid hormone sensitivity parameters were categorized into quartiles/quintiles, the ORs for having MetS at Q3, Q4 versus Q1 FT3 were 1.39 (95% CI: 1.14–1.70, *p* = 0.001) and 1.38 (95% CI: 1.13–1.69, *p* = 0.002), respectively. However, the OR for having MetS in the Q4 group versus the Q1 FT4 group was 0.71 (95% CI: 0.58–0.87, *p* = 0.001). The higher the TSH quartile was, the greater the risk of MetS (Q2: OR = 1.31, 95% CI: 1.08–1.60; Q3: OR = 1.36, 95% CI: 1.12–1.66; Q4: OR = 1.50, 95% CI: 1.23–1.83). Similar results were observed for TSHI, TT4RI and the FT3/FT4 ratio. Considering that the values of the TFQI and PTFQI range from −1 to 1, a value of 0 indicates normal sensitivity. We categorized them into quintiles and set Q3 as the reference group. The results revealed that there was no difference in the risk of MetS among the groups (Figure 3).

## 4 Discussion

In this cross‐sectional study, we found that increased FT3 levels and higher FT3/FT4 ratios were associated with increased risks of abdominal obesity, hypertension, and MetS. Elevated TSH and decreased FT4 levels were correlated with MetS and its individual components. In addition, reduced central thyroid hormone sensitivity and increased peripheral thyroid hormone sensitivity were identified as risk factors for MetS and related metabolic abnormalities in older Chinese adults.

The prevalence of MetS in our community‐dwelling older population from western China was 40.31%. Previous systematic reviews reported that the prevalence of MetS in Asian adults ranges from 11.9% to 49.0% [23]. While the CHARLS reported an overall MetS prevalence of 33.38% [4], our findings are more consistent with those of the Pinggu Metabolic Disease Study, which reported a prevalence of 42.8% in a euthyroid Chinese population [7]. Similarly, a study conducted in rural Northeast China reported a MetS prevalence of 39.0% using modified ATP III criteria [24]. Despite differences in diagnostic criteria and study designs, the MetS prevalence generally increases with age and is greater in women [4, 24]. Given that our cohort consisted of older individuals with a greater proportion of females than the CHARLS population did, the relatively higher prevalence observed in our study is reasonable.

Thyroid hormones are pivotal regulators of energy metabolism, thermogenesis, and glucose and lipid homeostasis, and their dysregulation predisposes individuals to metabolism‐related disorders. Hypothyroidism, characterized by a hypometabolic state, is associated with reduced energy expenditure, weight gain, dyslipidemia, impaired lipolysis, and disrupted gluconeogenesis, thereby increasing the risk of obesity, diabetes, and MetS [17]. Consistent with these findings, we observed that elevated TSH and decreased FT4 levels were significantly associated with MetS and its components. Hyperthyroidism also has substantial metabolic and cardiovascular effects. In fact, both hyper and hypothyroidism are established risk factors for hypertension, although their mechanisms differ: hypothyroidism is thought to increase peripheral vascular resistance, whereas hyperthyroidism primarily induces a hyperdynamic circulatory state [25, 26]. In our study, higher FT3 levels were positively correlated with increased WC, elevated BP and increased risk of MetS. These findings align with those of previous population‐based studies. For example, a study of euthyroid American adults reported that BMI and WC were positively correlated with serum TSH and FT3 levels but not with FT4 [27]. Another study among the oldest‐old in China demonstrated that FT3, T3, and the FT3/FT4 ratio were strongly positively associated with BMI and the waist‐to‐height ratio, whereas FT4 showed inverse associations [28]. Similarly, a large cross‐sectional study in a euthyroid Chinese population reported higher FT3 and FT4 levels in overweight and obese individuals [29]. Although the mechanisms linking obesity phenotypes to altered thyroid hormone profiles remain incompletely understood, proposed explanations include reduced thyroid hormone receptor expression in adipocytes, lipotoxicity‐induced thyroid hormone resistance, an altered sex hormone milieu, and insulin resistance [29, 30]. Previous studies have suggested that individuals with higher fat mass and poorer metabolic profiles may have upregulated peripheral deiodinase activity, possibly driving higher FT4‐to‐FT3 conversion [31]. In addition, elevated FT3 levels in individuals with abdominal obesity may reflect a compensatory increase in peripheral thyroid hormone activation aimed at promoting energy expenditure, lipolysis, and sympathetic nervous system activation [32]. In summary, both hypo‐ and hyperthyroid states may contribute to metabolic dysfunction. Therefore, screening thyroid function in individuals at high risk of MetS may facilitate early intervention.

Reduced sensitivity to thyroid hormones is a prevalent phenomenon in older adults and is implicated in metabolic disorders. Compared with isolated hormone measurements, composite indices of thyroid hormone sensitivity offer a more integrative assessment of thyroid homeostasis, providing a valuable clinical perspective on the upstream alterations in endocrine regulation and metabolic adaptability in older individuals with heterogeneous metabolic phenotypes [33]. Thyroid hormone resistance can be broadly classified into central and peripheral components. Previous studies have demonstrated that reduced central thyroid hormone sensitivity is associated with diabetes, hypertension, and MetS [7, 34, 35]. In agreement with these findings, we observed that reduced central sensitivity was significantly associated with hypertriglyceridemia, hypertension, and MetS in older adults. Potential mechanisms include leptin resistance, chronic low‐grade inflammation, impaired central thyroid hormone transport, and alterations in TR isoforms. Reduced central thyroid hormone sensitivity has also been linked to increased adipose tissue IR in euthyroid individuals with obesity [36]. However, although most studies reported significant associations between the TFQI/PTFQI and obesity, hypertension, diabetes, and MetS [20, 21, 35], our results demonstrated a significant association with hypertension but not with MetS. This discrepancy suggests that the metabolic implications of the TFQI/PTFQI may differ by age group and warrants further investigation in older populations.

FT3 and FT4 represent the circulating active forms of T3 and T4, and the peripheral conversion of T4 to T3 is mediated primarily by deiodinase activity. The FT3/FT4 ratio is therefore commonly used as an indicator of peripheral thyroid hormone sensitivity and deiodinase activity. In this study, an elevated FT3/FT4 ratio was associated with abdominal obesity, hypertriglyceridemia, hypertension, and MetS in older euthyroid individuals, even after adjustment for multiple confounders. These findings are consistent with previous studies showing that a higher FT3/FT4 ratio is associated with insulin resistance, adverse metabolic profiles, and MetS [13, 14, 37]. Elevated FT3/FT4 ratios have also been linked to inflammatory markers, arterial stiffness, and obesity‐related phenotypes [29, 38], all of which are characteristics of metabolic syndrome. However, there are some contradictions in the literature, with some studies reporting an inverse correlation between the FT3/FT4 ratio and metabolic disorders [7, 15].

The divergent associations reported in the literature may stem from several factors, particularly in older populations. First, the physiological interpretation of the FT3/FT4 ratio is complex in aging. Age‐related alterations in hypothalamic–pituitary–thyroid axis regulation, together with chronic low‐grade inflammation and changes in nutritional status, can substantially influence peripheral T4‐to‐T3 conversion independently of intrinsic thyroid function [39]. Second, the high prevalence of nonthyroidal illness syndrome among older adults further complicates interpretation, as this condition is characterized by reduced FT3 levels and altered FT3/FT4 ratios [40]. Moreover, a key explanatory factor may be the age‐related shift in thyroid hormone metabolism itself. Large‐scale studies indicate that peripheral conversion of FT4 to FT3 declines after midlife [41], and long‐lived individuals often exhibit lower FT3 and FT3/FT4 ratios alongside higher TSH—a pattern widely considered a protective, hypometabolic adaptation [28, 42, 43]. Therefore, an elevated FT3/FT4 ratio in older adults may signify a disruption of this adaptive state, potentially linking it to metabolic dysregulation. This is supported by evidence that higher FT3 levels and FT3/FT4 ratios are closely associated with insulin resistance, a central driver of MetS [44]. Taken together, although the FT3/FT4 ratio is commonly used to assess peripheral thyroid hormone sensitivity in cohort studies, FT3 concentrations in older populations are subject to multiple age‐related influences. Consequently, existing cross‐sectional findings require validation in longitudinal studies to better elucidate temporal and causal relationships.

Several limitations of this study should be acknowledged. First, thyroid‐related biochemical indices may be influenced by multiple factors, and a single measurement may not fully reflect long‐term thyroid hormone dynamics. Although we adjusted for a broad range of confounders, residual confounding cannot be entirely excluded. Second, we did not measure total thyroid hormones or thyroid autoantibodies, and the relationships between other thyroid hormone sensitivity indicators and MetS warrant further investigation. Third, the cross‐sectional design precludes causal inference, and the generalizability of our findings and the predictive value of these indices for MetS require validation in large‐scale longitudinal studies.

## 5 Conclusion

In conclusion, we found that abnormal FT3 levels, increased TSH levels, and decreased FT4 levels were correlated with MetS and its components in community‐dwelling older adults in western China. Reduced central thyroid hormone sensitivity and increased peripheral thyroid hormone sensitivity are risk factors for MetS and its components.

## Author Contributions

All authors made significant contributions and followed the latest guidelines of the International Committee of Medical Journal Editors (ICMJE). Fengjuan Hu, Hu Liu and Linlin Gu are co‐first authors. Fengjuan Hu and Birong Dong conceptualized this study and designed the study protocol; Fengjuan Hu, Hu Liu, Linlin Gu, Shuli Jia, Lixing Zhou, Wanyu Zhao, Xiaolei Liu performed the data collection and extraction; Fengjuan Hu and Hu Liu performed the statistical analyses; Fengjuan Hu, Hu Liu and Linlin Gu prepared the outlines and wrote the manuscript. All authors contributed to critical revision of the manuscript.

## Funding

This work was supported by the National Natural Science Foundation of China (No. 92248304, PI: B.D.), the Natural Science Foundation of Sichuan Province, China (No. 2024NSFSC1604, PI: L.Z.), the Sichuan Science and Technology Program (No. 2025ZNSFSC1597, PI: X.L.), Project of Sichuan Province Health Commission (24LCYJPT01, PI: X.L.), National Clinical Research Center for Geriatrics, West China Hospital, Sichuan University (Z2023JC005, PI: W.Z.; Z2023LC005, PI: L.Z.; Z2024JC008, PI: X.L.), Project of Chengdu Science and Technology (2024‐YF05‐02255‐SN), 1·3·5 project for disciplines of excellence‐Clinical Research Fund, West China Hospital, Sichuan University (2025HXFH040). The financial sponsors had no role in the design, implementation, analysis, or reporting of the results.

## Ethics Statement

The WCHAT study was registered in the Chinese Clinical Trial Registry (registration no. ChiCTR1800018895) and was approved by the Ethics Committee of West China Hospital, Sichuan University (approval no. 2017‐445). This study was conducted in accordance with the Declaration of Helsinki, and written informed consent was obtained from all participants and/or their proxy respondents.

## Conflicts of Interest

The authors declare no conflicts of interest.

## Data Availability

Data in this article are confidential and not publicly available. But those data are available from the corresponding author upon a reasonable request.
